# Consuming alternative prey does not influence the DNA detectability half-life of pest prey in spider gut contents

**DOI:** 10.7717/peerj.7680

**Published:** 2019-10-22

**Authors:** Dávid Fülöp, Éva Szita, Regina Gerstenbrand, Gergely Tholt, Ferenc Samu

**Affiliations:** Department of Zoology, Plant Protection Institute, Centre for Agricultural Research, Budapest, Hungary

**Keywords:** Spider natural enemy, DNA detectability half-life, Digestion time, PCR, Cereal pest, Leafhopper

## Abstract

**Background:**

Key natural enemy-pest interactions can be mapped in agricultural food webs by analysing predator gut content for the presence of a focal pest species. For this, PCR-based approaches are the most widely used methods providing the incidence of consumption of a focal pest in field sampled predators. To interpret such data the rate of prey DNA decay in the predators’ gut, described by DNA detectability half-life (*t*_1/2_), is needed. DNA decay may depend on the presence of alternative prey in the gut of generalist predators, but this effect has not been investigated in one of the major predatory arthropod groups, spiders.

**Methods:**

In a laboratory feeding experiment, we determined *t*_1/2_ of the key cereal pest virus vector leafhopper *Psammotettix alienus* in the digestive tracts of its natural enemy, the spider *Tibellus oblongus*. We followed the fate of prey DNA in spiders which received only the focal prey as food, or as an alternative prey treatment they also received a meal of fruit flies after leafhopper consumption. After these feeding treatments, spiders were starved for variable time intervals prior to testing for leafhopper DNA in order to establish *t*_1/2_.

**Results:**

We created a PCR protocol that detects *P. alienus* DNA in its spider predator. The protocol was further calibrated to the digestion speed of the spider by establishing DNA decay rate. Detectability limit was reached at 14 days, where c. 10% of the animals tested positive. The calculated *t*_1/2_ = 5 days value of *P. alienus* DNA did not differ statistically between the treatment groups which received only the leafhopper prey or which also received fruit fly. The PCR protocol was validated in a field with known *P. alienus* infestation. In this applicability trial, we showed that 12.5% of field collected spiders were positive for the leafhopper DNA. We conclude that in our model system the presence of alternative prey did not influence the *t*_1/2_ estimate of a pest species, which makes laboratory protocols more straightforward for the calibration of future field data.

## Introduction

Natural enemies exert an important controlling function in agricultural food webs, as predation on pests may have a beneficial cascading effect on crop plants (Mooney et al., 2010). Generalist predators, like spiders or carabid beetles, usually target a wide spectrum of prey species which makes it difficult to determine their effective participation in various trophic pathways. Currently, it remains an important task to identify key natural enemies that may have the greatest effect in the reduction of herbivorous pest insects.

Mapping trophic interactions in the nature can be a demanding endeavour, especially considering small species with hidden lifestyle. In predatory insects with chewing mouthparts, where physical traces of the prey might be detectable in the digestive system, gut content dissection could provide data about prey identity (Sunderland et al., 1987). However, in spiders extra-oral digestion and the exclusive consumption of liquidised food precludes traditional gut content analysis. To make prey detection easier, in the past decades DNA-based methods (Greenstone & Shufran, 2003; Symondson, 2002) have been developed and applied extensively.

Among the ever wider array of molecular methods, if a specific trophic relationship needs to be screened, polymerase chain reaction (PCR) based methods still dominate and seem to be the most practical and cost-effective solution (Symondson & Harwood, 2014). However, to be able to interpret molecular results obtained from field sampled animals in an ecologically meaningful way, the temporal pattern of the detectability of prey DNA has to be established (Athey, Sitvarin & Harwood, 2017; Greenstone et al., 2014; Sint et al., 2011). Prey DNA is digested in predator intestines with variable speed, therefore calibrating how long prey is detectable is crucial for the ecological interpretation of PCR results. For this, DNA detectability half-life (*t*_1∕2_)—defined as the time after feeding at which prey remains could be detected in only half of the assayed predators—is a widely accepted measure (Greenstone et al., 2014).

Spiders show considerably high variation in detectable prey DNA half-lives (Kobayashi et al., 2011). Spiders are adapted to huge variations in prey availability (Samu & Bíró, 1993; Wise, 1993). They are able to starve for long periods, which is aided by lowered metabolic rates (Tanaka & Ito, 1982), and also by the storage of ingested food in their branching midgut (Foelix, 2010). Most spiders are highly generalist predators that consume alternative prey even if high density of a particular suitable prey species is present (Harwood, Sunderland & Symondson, 2004). Molecular gut content analysis in spiders has proven that while consumption of multiple prey species is common, consumption frequency does not directly reflect the field abundance of the various prey (Kuusk & Ekbom, 2012; Whitney et al., 2018). For this reason, it is important to assess actual field predation rates, such as between our focal species, the significant virus vector cereal pest *Psammotettix alienus* (Dahlbom, 1850) and its natural enemy, the agrobiont spider *Tibellus oblongus* (Walckenaer, 1802).

*Psammotettix alienus* (Auchenorrhyncha, Cicadellidae) is one of the most abundant leafhopper pest species in cereals in the Holarctic region. It is an oligophagous herbivorous insect with host plants belonging to the Poaceae family. It is mostly found in cereal fields and in grassy field margins. *Psammotettix alienus* feeds on the phloem sap of its host plants (Tholt, Samu & Kiss, 2015). Direct damage caused by the feeding is negligible; however, *P. alienus* can cause significant losses in cereals by being the only vector of the Wheat Dwarf Virus (WDV) (Nygren et al., 2015). As WDV is a phloem related circulative, non-propagative virus, its inoculation and transmission depends on the phloem feeding of its vector (Tholt et al., 2018). Since phloem is embedded deep in leaf tissues, and the withdrawal of mouth parts from here requires more time than from more superficial tissues (Tholt et al., 2018; Zhao et al., 2010), phloem feeding makes these vector insects highly vulnerable to predators.

*Tibellus oblongus* (Araneae, Philodromidae) is the most abundant plant-dwelling non-web-weaver spider in Central European agro-ecosystems (Samu & Szinetár, 2002). In our previous studies we have provided evidence that this spider species is potentially an important predator of *P. alienus* (Samu, Beleznai & Tholt, 2013). Besides predation, its presence caused important non-consumptive effect (NCE) on the leafhopper leading to delayed and reduced sap feeding (Beleznai et al., 2015; Samu, Beleznai & Tholt, 2013; Tholt et al., 2018). In spite of laboratory feeding trials and massive field co-occurrence data, traditional sampling methods could not directly prove that *T. oblongus* regularly preys on *P. alienus* in the field. DNA based gut content analysis could provide such an evidence.

Since in generalist predators, such as spiders, concurrent digestion of alternative prey may change digestion and detectability of focal prey DNA (Greenstone et al., 2014), any calibration attempt to establish *t*_1∕2_ should take this factor into account. Few studies consider how alternative “chaser” prey, fed after the focal prey, affects focal prey DNA digestion in insects (e.g.,  Weber & Lundgren, 2009). We are not aware of any comprehensive study that addresses the effect of the presence of alternative prey on the results of molecular gut content analysis in spiders. Therefore, in spiders in general, and in the specific case of our focal species, it remains to be shown and quantified how DNA detectability half-life is affected when more than one prey type is digested by the spider.

In this study we assess the trophic connection between *P. alienus* and *T. oblongus.* We achieve this (i) by creating and testing a PCR reaction that detects the presence of leafhopper DNA in the spider; (ii) by calibrating this reaction to the digestion speed of the spider through the establishment of *t*_1∕2_ value; (iii) by extending the calibration to scenarios when alternative prey or no alternative prey was made available after the predation event on the focal prey; (iv) test the applicability of the PCR reaction on spiders collected from fields with *P. alienus* infestation.

## Materials & Methods

### Focal species

*Tibellus oblongus* specimens were collected from field margins in the vicinity of our field station at Nagykovácsi, Hungary (N47°32′52.81″E 18°56′1.15″) by using a sweep net. Collected spiders entered our stock population. Spiders in the stock population were housed separately in transparent plastic containers (30 mm diam., 67 mm high), with a layer of moistened plaster of Paris at the bottom. The containers were kept in climate chamber under 22 °C and 16:8 (L:D) photoperiod. Spiders were fed with *Drosophila melanogaster* Meigen, 1830 adults (size range 2–4 mm, mass reared on an agar-based media at room-temperature) once a week. For feeding, more *Drosophila* specimens were offered than the spiders could consume in one meal. Spiders were kept on this diet for at least three weeks prior to any experimentation.

The focal prey, the leafhopper *P. alienus*, was also kept as a stock population, reared under 24 °C and 16:8 (L:D) photoperiod on potted tillering barley plants (*Hordeum vulgare* L.) (Tholt et al., 2018; Tholt, Samu & Kiss, 2015). The stock population was periodically refreshed form cereal fields nearby Perbál, Hungary (N47°34′30.87″E 18°45′59.63″). Field collected animals were quarantined for one generation before entering stock population. Adult individuals (size range: 4–5 mm) were selected randomly for feeding trials.

### Laboratory feeding trials

The experiment was carried out in three trials. In each feeding trial large juvenile and subadult spiders (body length range: 7–11 mm) were selected from the stock. Before entering the trials the selected spiders were starved for one week in a clear container and then were randomly assigned to treatments. In the “leafhopper” (L) feeding treatment, spiders only received one leafhopper at time 0 h as prey; in the “leafhopper + *Drosophila*” (LD) treatment, one leafhopper prey was given the same way and three additional fruit flies were offered at time 24 h (Table 1). With this protocol we intended to simulate the effect of a “next prey item”. Previous data suggests agricultural field spiders feed an average of once a day (Nyffeler & Breene, 1990), which is reinforced by a recent literature survey on volumetric daily prey consumption of spiders (Nyffeler & Birkhofer, 2017). Feeding activity was observed visually. The time window allowed for feeding was 2 h. Spiders which did not consume the prescribed prey (either focal or alternative) in 2 h, were excluded from the experiment. After feeding, spiders were transferred to clear containers to avoid any contact with food remnants, and starved for the digestion periods pre-set for the given trial. At the end of the pre-set digestion period, spiders were placed in a sterile microcentrifuge tube, killed instantly on dry ice, and stored at −22 °C for later use.

**Table 1:** Experimental setup of feeding trials.

		**Digestion period (hours)**							
**Trial**	**Treatment**	**0**	**8**	**24**	**48**	**96**	**144**	**192**	**336**
I.	L	+L	3		10	10	10	10	
	LD	+L	3	+D	10	10	10	10	
II.	L	+L	5		10	10	10	10	
	LD	+L	5	+D	10	10	10	10	
III.	L	+L							19
	LD	+L		+D					20

**Notes.**

+L leafhopper food received +D Drosophila food received numbers sample size (number of spiders killed and tested at the end of the given digestion period)

The pre-set digestion periods were timed as multiples of full days (24 h), except for a first period of 8 h after feeding, which served as a positive control. To keep synchrony between the treatment groups, the first non-control samples from the treatment populations were taken on day two, 24 h later than LD spiders received *Drosophila* meal. Overall, in the first two trials (*N* = 176 spiders) digestion periods were set at 8 h, 2, 4, 6 and 8 days. In the third trial (*N* = 39 spiders) we aimed to study longer term digestion, and the only digestion period implemented was 14 days. Pre-set digestion periods per trials and treatments with sample sizes per period are given in Table 1.

### Field applicability trial

To test whether our PCR protocol can be used to determine if field collected spiders contain *P. alienus* DNA, a small field survey was conducted on 10.10.2018. In self-sown wheat (same fields near Perbál, from where stock population originates), with hand collection, we collected *N* = 40 *T. oblongus* individuals. These post-harvest fields with self-sown cereal had a modest *P. alienus* density, meaning that when walking on the field, a number of jumping *Psammotettix* individuals was regularly visible. Species identity was checked from sweep net samples, but leafhopper density was not determined explicitly. Each collected spider individual was immediately killed on dry ice, transferred and stored in a refrigerator. These individuals were tested for the presence of *Psammotettix* DNA the same way as individuals from the laboratory feeding trials.

### Molecular methods

Whole spider specimens were used to extract DNA with Extraction Solution and Dilution Solution (Sigma-Aldrich, Saint Louis, MO, USA) according to manufacturer protocol. All samples were macerated using sterilised pipette tips (Chum et al., 2012). Ten taxon specific primer pairs, amplifying 200–300 bp long segment of mitochondrial cytochrome-oxidase I (COI) barcoding region was designed using the NCBI Primer-blast home page (http://www.ncbi.nlm.nih.gov/tools/primer-blast/) following the recommendations of King et al. (2008) and tested in silico for *Psammotettix* specificity against GenBank nucleotid database of Arthropoda taxa with the same application. After optimisation we chose the Psam268F (5′-ACCACCATCTATCACCCTACT-3′) and Psam483R (5′-CATACAAATAATGGTGTGCG-3′) primer pair for further application.

All PCR reactions were carried out on Arktik Thermal Cycler (Thermo Fisher Scientific, Waltham, MA, USA), tubes containing 5 µl 5x HOT FIREpol^®^ Blend Master Mix with 10 mM MgCl_2_ (Solis BioDyne, Tartu, Estonia), 0.25 µl of each primer (c. 10 mM), 2 µl template DNA and 17.5 µl MQ water. The cycling conditions were 15 min at 95° C, 35 cycles of 30 s at 95 °C, 30 s at 54 °C, 1 min at 72 °C and final elongation of 5 min at 72 °C.

To avoid cross contamination, sterile filter tips were used in all pipetting steps. Every PCR reaction was carried out within 1-3 days after DNA extraction. In every PCR run one negative control was used, containing only the PCR mastermix with MQ water. As negative controls, in separate PCR runs, we have also tested our primers on non-target *Drosophila* and *Tibellus*, using *Psammotettix* DNA as positive control. Since no non-target amplification was observed, these controls were not added to later runs.

The PCR product was visualised by agarose gel electrophoresis on MLB16 UltraBright UV Transilluminator (Maestrogen, Hsinchu, Taiwan). Amplification was taken as positive if clear band of expected size was visible with the naked eye, regardless of the intensity of the band.

In the case of *T. oblongus* specimens collected in the field applicability trial, PCR positive products were purified from the gel using GenElute™ PCR Clean-Up Kit (Sigma-Aldrich, St. Louis, MO, USA) following manufacturer protocol. Sanger sequencing were carried out by Macrogen Europe (The Netherlands) using the same primer pair. Sequences were checked and edited by Staden package (Staden, Beal & Bonfield, 2000) and MEGA7.0.26 (Kumar, Stecher & Tamura, 2016) software. We asserted that these sequences taxonomically represent the species *Psammotettix alienus* via nblast search in GenBank (http://www.ncbi.nlm.nih.gov/blast/Blast.cgi) and via the construction of phylogenetic networks in SplitsTree v.4.14.6 (Huson & Bryant, 2005) using the sequences taken from a phylogenetic study dealing with *Psammotettix* populations of Europe (Abt et al., 2018).

### Statistical methods

Data were analysed with generalised linear models (GLM, binomial distribution, logit link). Akaike information criterion was employed to choose between models using stepwise model selection. Molecular detectability half-life (*t*_1∕2_) was calculated using the best fitted model. All calculations were executed with PAST3.23 (Hammer, Harper & Ryan, 2001) and R version 3.5.1 (R Development Core Team, 2018) programs.

## Results

### Primer

With the Psam268F and Psam483R primer pair a 215 bp long segment of the target gene (COI) was amplified. No unspecific amplicon was observed after laboratory optimisation, including testing on control starved *T. oblongus* and on *D. melanogaster*. All sequences are available in GenBank under accession numbers: MK471356, MK491183 –MK491187.

### Laboratory feeding trials

**Figure 1:**
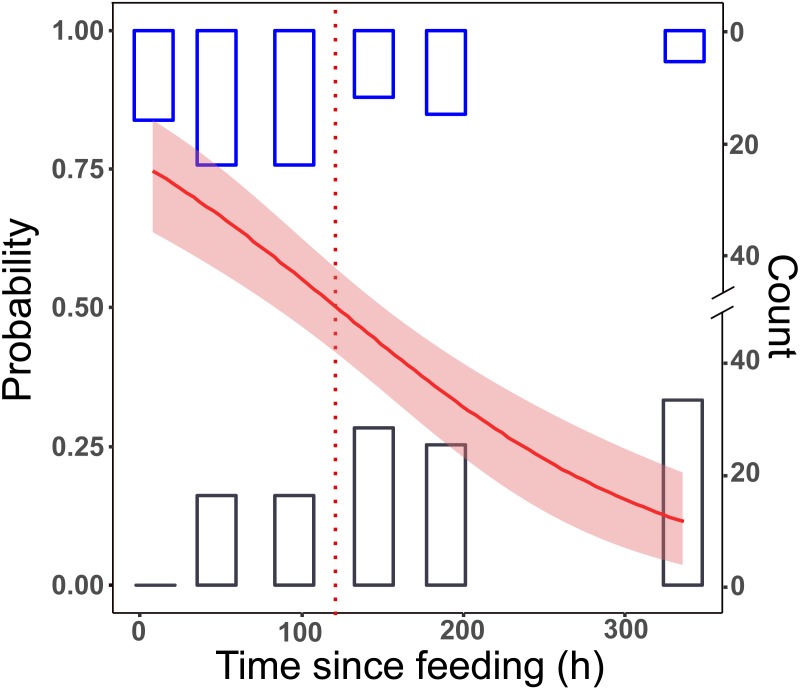
Detection of prey DNA in *T. oblongus* specimens’ gut content with diagnostic PCR reaction. Probability of detection was estimated with GLM. The number of specimens tested as positive or negative are represented as histograms. Red line: fitted logit model with standard error (pink); dotted line: *t*_1∕2_, bars: cumulative number of positive (blue) and negative (black) PCR reactions at the given time point in the three feeding trials (see Table 1 for details of trials).

All samples tested positive after 8 h. This dropped to 65% in group L, and to 55% in group LD for the 48 h post-feeding samples. After this, we received positive PCR results in a decreasing manner. At the longest detection period, after 14 days, 10% of the samples were PCR positive (Fig. 1).

No significant differences between the first two trials (differing only in the date of execution) could be detected (effect of trial: GLM, binomial distribution, logit model, *z* = 0.357, *p* = 0.72), therefore the trial effect was not included in further models. The final logit model indicated that the detectability of focal prey DNA significantly decreased with time (Fig. 1), but the two feeding treatments, whether alternative prey was consumed or not, did not affect the rate of this decrease (Table 2). Molecular detectability half-life was estimated on the simplified model with time as the only predictor, resulting in an estimate of nearly exactly 5 days (*t*_1∕2_ = 121.6 h ± 15.84 h).

**Table 2:** The effect of digestion time and alternative prey consumption and the detectability of *Psammotettix alienus* DNA in *Tibellus oblongus* gut content. Generalised linear model with binomial distribution and logit link.

		**Estimate**	**Standard error**	*z* value	*P* value
Time since feeding		−0.011	0.003	−4.085	>0.0001
Treatment (consuming alternative prey)		−0.588	0.558	−1.054	0.2918
Time since feeding and treatment interaction		0.002	0.004	0.571	0.5677

### Field applicability trial

The primer pair tested positive in five out of 40 field collected spiders. All positive samples were sequenced and identified as *P. alienus*.

## Discussion

Our laboratory results demonstrated that focal prey DNA traces were detectable in the digestion system of the generalist spider predator *T. oblongus* with a considerable length of 5 days DNA detectability half-life, irrespective of whether alternative prey was also digested. Two weeks after feeding c. 10% of the spiders were positive for focal prey DNA, indicating that this time span is the approximate limit of DNA detectability in our model predator and testing system. The applicability of this molecular gut content analysis protocol was proven in a field trial, where 12.5% of the field collected *T. oblongus* individuals tested positive for *Psammotettix* DNA.

While traditional protein-based molecular methods indicated that, as compared to other predatory groups, prey remains are detectable in spiders for relatively long time periods (Sunderland et al., 1987), *t*_1∕2_ reports give rather variable figures for spiders. Some studies estimate as short durations as few hours, such as in spiders feeding on the Russian wheat aphid, *Diuraphis noxia* (*Tetragnatha* sp.: *t*_1∕2_ = 4.2 h, *Pardosa* sp.: *t*_1∕2_ = 2.0 h) (Kerzicnik et al., 2012), or in a lynx spider (Oxyopidae) feeding on stink bugs (*t*_1∕2_ = 8.2 h) (Athey, Sitvarin & Harwood, 2017). However, other detectability half-life studies in spiders give at least one order of magnitude higher estimates. Examples include *t*_1∕2_ = 83 h in *Dysdera* sp. (Macias-Hernandez et al., 2018); in multiple spider species *t*_1∕2_ = 36–192 h in the study of Kobayashi et al. (2011); and *t*_1∕2_ = 204–516 h reported by Pompozzi et al. (2019). In this respect, our *t*_1∕2_ estimate of 121 h for *T. oblongus* can be regarded as typical within spiders.

Such a variation in *t*_1∕2_ might be related to the fact that taxonomically different spider groups with different feeding strategies have been examined, even though Pompozzi et al. (2019) found only minimal difference between DNA decay rate in specialist versus generalist spiders. One of the shortest *t*_1∕2_ values were reported for the lynx spider *Oxyopes salticus,* in which the trial spiders were willing to feed on the offered brown marmorated stink bugs only after considerable starvation in laboratory arenas, and completely refused the prey in mesocosm enclosures (Athey, Sitvarin & Harwood, 2017). This raises the possibility that prey retention in spiders’ digestive tracts may depend on the preferential status of the prey, i.e., non-preferential prey is purged out sooner. Detectability half-life estimates have also been shown to depend on temperature during testing (Kobayashi et al., 2011; Von Berg et al., 2008); the type of sample from the animal (Kamenova et al., 2018; Macias-Hernandez et al., 2018); external DNA contamination and other artefacts related to field sampling (Greenstone et al., 2012; King et al., 2012). However, many, if not all of these factors can be eliminated, standardised or controlled for during the establishment of *t*_1∕2_.

A further important candidate factor in generalist predators, that might need to be controlled for, is the consumption of alternative prey. Spiders are regarded to be generalist predators, even if some taxa show significant level of prey specialization (Pekar, Coddington & Blackledge, 2012). Even the non-specialist spiders, such as our study object, *T. oblongus*, may choose preferentially from the available prey spectrum (Whitney et al., 2018). It seems to be the norm that these predators, even if one of their preferential prey species is abundant, consume alternative prey species, as well (Kuusk & Ekbom, 2012). Concurrent digestion of alternative prey may significantly alter focal prey DNA detectability half-life in arthropod predators (Greenstone et al., 2014). Such a significant “chaser prey” effect was found in the coccinellid predator *Coleomegilla maculata* (Weber & Lundgren, 2009), but this predator radically differs in feeding mode and digestion dynamics from spiders. In spiders, we are aware of two studies that address the question of multiple prey digestion. Due to the different focus of the paper, chaser prey effect was not compared to no-chaser prey control in a study on the woodlouse hunter spider *Dysdera verneaui* (Macias-Hernandez et al., 2018). In another study, aiming at the detection of *Plutella xylostella* (Lepidoptera) DNA in the gut of the wolf spider *Venator spenceri*, chaser prey effect was only investigated after a fixed period of starvation (Hosseini, Schmidt & Keller, 2008). In the present study the consumption of the focal prey was followed by the consumption of an alternative prey item. The two instances were separated by 24 h, a period that follows from the general estimate of predation frequency of spiders (Nyffeler & Birkhofer, 2017; Nyffeler & Breene, 1990). Under these circumstances the concurrent digestion of alternative prey did not affect the detectability half-life of the focal prey species.

Estimating the frequency of predator individuals positive for a given prey, jointly with the detectability half-life of prey DNA, make it possible to obtain an estimate of the minimal predation frequency on a given prey, and thus discover and weight food web links important for biological control (Greenstone et al., 2014; Roslin & Majaneva, 2016). When testing for the presence of *Psammotettix* DNA in *T. oblongus* with PCR and subsequent sequencing, we primarily wanted to show that the laboratory protocol works on field collected predators. Out of the 40 specimens examined, 5 tested as positive. However, due to the lack of monitoring prey density, and the one time sample, we do not attempt to interpret this 12.5% positive result in the context of the predatory efficiency of spiders. Where such interpretations were attempted, variable prey-positivity of spiders was found. When testing several taxa, on average only 17% of the predator individuals were positive for the rosy apple aphid (*Dysaphis plantaginea*) during its peak infestation period, although here the best performing taxa showed values above 30% (Lefebvre et al., 2017). The highest reported positivity reported for a spider was 71% for *Pardosa* spp. individuals preying on the aphid *Rhopalosiphum padi*, when aphid density at ground level was as high as 60 individuals per 50 cm^2^ (Kuusk & Ekbom, 2012).

While high prey density obviously seems to raise prey DNA positivity in predators, the sedentary or more agile nature of prey also appears to be an important factor, especially for non-web building spiders. Lower positivity figures were published for more motile prey. For instance, for the sharpshooter *Homalodisca vitripennis* in citrus groves only 1.97% of spiders were found positive with PCR (Hagler et al., 2013). DNA of the moderately motile pollen beetle (*Meligethes aeneus*) was found by PCR detection in 13.8% of the tested hunting spider (*Pardosa* sp.), but in much higher percentage (51.7%) of the other tested spider species, *Theridion impressum*, which catches its prey with web (Öberg, Cassel-Lundhagen & Ekbom, 2011). Non-consumptive effects may also add to the effectiveness of spider predation. Just in the tested *Psammotettix*-*Tibellus* model system, in previous studies, we have found that the mere presence of spiders delays and shortens feeding in the leafhopper, thus reducing the vectoring efficiency of the plant virus (WDV) it spreads (Beleznai et al., 2015; Tholt et al., 2018). These studies highlight that in order to comparatively assess the efficiency of arthropod predators based on molecular gut content analysis results, many factors, among others prey density, prey agility and mode of predation, are needed to be taken into account.

## Conclusions

Our study indicates that one important factor, the presence of alternative prey in the gut of spiders, which is highly applicable to field populations, did not distort laboratory DNA half-life measurements. Thus, results obtained from the simplified single prey type protocol, if other conditions are controlled for, might be applicable to calibrate field results.

##  Supplemental Information

10.7717/peerj.7680/supp-1 Supplemental Information 1 Results of PCR reactions

## Additional Information and Declarations

### DNA Deposition

The following information was supplied regarding the deposition of DNA sequences: All sequences are available in GenBank: MK471356, MK491183 –MK491187.

### Data Availability

The following information was supplied regarding data availability: The results of the PCR reactions are available in the Supplemental File.
